# 

**Published:** 1948

**Authors:** 


### The Medical Library of the University of Bristol

WITH WHICH IS INCORPORATED

**The Library of the Bristol Medico-Chirurgical Society.**

---

*The following donations have been received since the publication of the list in the Last issue.*

| | |
|---|---|
| American College of Surgeons (1) .. .. .. | 1 volume. |
| Buenos Aires University (2) .. .. .. .. | 1 volume. |
| Calcutta University (3) .. .. .. .. .. | 2 volumes. |
| E. Wilfred Fish, Esq. (4) .. .. .. .. .. | 1 volume. |
| Iowa State Medical Society (5) .. .. .. .. | 3 volumes. |
| Michell Clarke Fund (6) .. .. .. .. .. | 2 volumes. |
| Mrs. C. A. Moore (7) .. .. .. .. .. | 22 volumes. |
| J. H. Perry, Esq. (8) .. .. .. .. .. | 1 volume. |
| Public Health Institute, Chicago (9) .. .. .. | 1 volume. |
| Dr. J. Samuels (10) .. .. .. .. .. | 1 volume. |
| J. E. Shaw Fund (11) .. .. .. .. .. | 1 volume. |
| Wilmer Ophthalmological Institute (12) .. .. | 1 volume. |
| Messrs John Wright & Sons, Ltd. (13) .. .. .. | 3 volumes. |

Unbound periodicals have also been received from the " Black Bag ", Dr. C. D. Evans, G. M. FitzGibbon, Esq., Professor R. Milnes Walker, and Messrs. John Wright & Sons, Ltd.

### THE ONE HUNDRED AND EIGHTY-NINTH LIST OF BOOKS.

The figures in round brackets refer to the figures after the names of the donors. The books to which no such figures have been attached have either been bought from the Library Fund or received through the *Journal.*

**Bailey, H. H.** .. .. *Emergency Surgery, Part* 2 .. .. .. 7th Ed. 1948

**Bailey, H. H. and Love, M.** *Short Practice of Surgery, Part I.* .. .. 8th Ed. 1948

**Bell, E. T.** .. .. .. *Renal Diseases* .. .. .. .. .. .. .. .. (6) 1946

**Bourne, A. W. and Williams, L. H.** *Recent Advances in Obstetrics and Gynaecology* 7th Ed. 1948

**Bowden, A. C.** .. .. *Essentials of Local Anaesthesia in Dentistry* (13) 1945

**Brahmachari, Sir U.** .. *Gleanings from my Researches* .. 2 Vols. (3) 1940–41

*British Pharmacopoeia* .. .. .. .. .. .. .. .. .. .. .. 1948

**Bronner, M.** .. .. .. *Dental Surgeon's Handbook* .. .. 2nd Ed. (13) 1948

**Carling, Sir E. R. and Ross, J. P. (Eds.)** *British Surgical Practice* Vol. 3 (11) 1948

**Cunningham, D. J.** .. *Textbook of Anatomy* .. .. .. 8th Ed. (6) 1947

**Currie J. R. and Mearns, A. G.** *Hygiene* .. .. .. .. .. 3rd Ed. 1948

**Daniels, M. and others** *Tuberculosis in Young Adults—Royal College of Physicians Prophit Tuberculosis Survey*, 1935–44 1948

**Dorland, W. A. M.** .. *American Illustrated Medical Dictionary* 21st Ed. 1948

**Duran, C. M.** .. .. *Las Ciencias Medicas en Guatemala* .. 2nd Ed. 1945

**Fish, E. W.** .. .. *Surgical Pathology of the Mouth* .. .. .. (4) 1948

**Gaddum, J. H.** .. .. *Pharmacology* .. .. .. .. .. .. 3rd Ed. 1948

**Gray, H.** .. .. .. *Anatomy—Descriptive and Applied* 24th Ed. (8) 1930

**Hale-White, W.** .. .. *Materia Medica, Pharmacy, Pharmacology and Therapeutics* .. .. .. .. .. 27th Ed. 1948

**Handfield-Jones, R. M. and Porritt, A. E.** *Essentials of Modern Surgery* 3rd Ed. 1948

**Hewer, C. L.** .. .. *Recent Advances in Anaesthesia and Analgesia* 6th Ed. 1948

**Hirsekorn, H.** .. .. *Denture Base Readjustment* .. .. .. .. (13) 1943

**Jennings, W. A. and Russ, S.** *Radon—its Technique and Use* .. .. .. 1948

**Maxwell, J.** .. .. .. *Care of Tuberculosis in the Home* .. .. 2nd Ed. 1948

**Molina, J.** .. .. .. *Cavidades para Incrustaciones de Resinas Acrilicas* (2) 1944

**Ogilvie, W. H.** .. .. *Forward Surgery in Modern War* .. .. .. (7) 1944

**Paterson, R.** .. .. *Treatment of Malignant Disease by Radium and X-rays* .. .. .. .. .. .. .. .. .. .. 1948

**Public Health Institute, Chicago** *How Laymen Cut Medical Costs* .. .. (9) 1948

**Samuels, J.** .. .. .. *Endogenous Endocrinotherapy including the Causal Cure of Cancer* .. .. .. .. .. .. .. (10) 1947

**Smout, C. F. Z. and Jacoby, F.** *Gynaecological and Obstetrical Anatomy* 2nd Ed. 1948

### TRANSACTIONS, REPORTS, JOURNALS, ETC.

American College of Surgeons' Year Book, 1947–1949 .. .. .. .. .. (1) 1947

Collected Reprints of the Wilmer Ophthalmological Institute. Vol. 8, 1946–1947 .. .. .. .. .. .. .. .. .. .. .. .. (12) 1948

International Clinics, 3rd Series, Vol. 4 ; 4th Series, Vols. 3, 4 (5) 1894–1895

Kongresszentralblatt fur Innere Medizin. Vols. 40–65 .. .. .. 1926–1932

Quarterly Cumulative Index Medicus. Vol. 41, Jan–June, 1947 .. .. .. 1948

Surgery, Gynecology and Obstetrics. Index Vols. 1–40 (1905–1925) .. .. 1926

Vols. 41–80 (1925–1945) .. .. 1948

### Publications Received

From Messrs. Angus & Robertson, Ltd., Sydney :

"*Elective Alimentary Rest*" *and the Elimination of so-called* "*Paralytic Ileus*" *after Abdominal Operations.* By V. J. Kinsella, M.B., Ch.M. (Syd.), F.R.C.S., F.R.A.C.S. Price 3s. 1948.

*The Mechanism of Abdominal Pain.* By V. J. Kinsella, M.B., Ch.M. (Syd.), F.R.C.S.(Eng.), F.R.A.C.S. Price 32s. 6d. 1948.

From Messrs. H. K. Lewis & Co. Ltd. :

*Infra-red Irradiation.* By William Beaumont, M.R.C.S.(Eng.), L.R.C.P.(Lond.) with a foreword by Lord Horder, G.C.V.O., M.D., F.R.C.P. Third Edition. Price 8s. 6d. 1948.

*Radiotherapy and Cancer.* By A. G. C. Taylor, M.R.C.S., L.R.C.P., D.R., F.F.R. and others. Price 7s. 6d. 1948.

*Psychiatric Case-taking Cards.* By A. Spencer Paterson. Second Edition. Price 9d. each. 1948.

From Dr. E. Henty Smalpage :

*The Universal Law of Chemical Decomposition.* By E. Henty Smalpage, M.B., Ch.M., F.R.C.S. 1948.

From Sylviro Publications, Ltd. :

*Handbook of Medicine for Final Year Students.* By G. F. Walker, M.D., M.R.C.P. Fourth Edition. Price 25s. 1948.

From Messrs. John Wright & Sons, Ltd. :

*British Journal of Surgery.* Vol. 36. No. 141. August 1948. Price 12s. 6d. (Annual Subscription 42s.). 1948.

*The Clinical Apprentice—a Guide for Students of Medicine.* By John M. Naish, M.D., M.R.C.P. and John Apley, M.D., M.R.C.P. Price 15s. 1948.

*Malignant Disease and its Treatment by Radium.* By Sir Stanford Cade, K.B.E., C.B., F.R.C.S. Second Edition. Vol. 1. Price 52s. 6d. 1948.

*Tuberculosis in Childhood.* By Dorothy Stopford Price, M.D. Second Edition. Price 25s. 1948.

*Demonstrations of Physical Signs in Clinical Surgery.* By Hamilton Bailey, F.R.C.S., F.A.C.S. Second Edition. Part 2. Price 8s. 6d. 1948.

### Local Medical Notes

On July 15th, 1948, The Stonebridge Press, the new printing works of Messrs. John Wright & Sons, Ltd., was formally opened by the Lord Mayor of Bristol, assisted by the Sheriff : one of the chief speakers at the luncheon afterwards was Sir Cecil Wakeley, Editorial Secretary to the *British Journal of Surgery*, one of the Company's best-known publications. In his speech he reminded his hearers that the *British Journal of Surgery* held an unrivalled position amongst surgical periodicals throughout the world, and that there was accordingly a tremendous demand for it wherever the Art of Surgery was practised. He deplored the fact that in spite of its intrinsic humanitarian worth and also of its value as an export, the policy of the *Journal* was still hampered by paper restrictions.

Mr. H. Chitty, Sheriff of Bristol, said that the publications of the House of Wright were renowned throughout the medical world : and the Lord Mayor, referring to " the vivid and serious enterprise " of the firm, said that he wished it ever-increasing success in its essential task of supplying medical textbooks to all quarters of the globe. Warm tributes were paid to the many printers in Bristol and the West of England who had helped the Company to maintain the technical excellence of its books during the seven years since the destruction of their Bristol factory, and also to the many work-people who had maintained their connection with the Company throughout.

Professor R. H. Parry, M.D., F.R.C.P., has been elected President of the Society of Medical Officers of Health.

### EXAMINATION RESULTS.

**University of Bristol.**—Students of the University have recently passed the following examinations :

M.B., Ch.B.—*Supplementary First Examination :* J. Laltoo, A. J. MacDonald, Catherine M. Stephen.

B.D.S.—*Supplementary First Examination :* P. Crane, T. F. Harris.

L.D.S.—*Supplementary First Examination :* C. G. Innocent, D. McCandlish, D. R. Salter.

L.D.S., R.C.S.—*Final Examination. Part II :* R. J. P. Cousins.

M.R.C.S., L.R.C.P.—*Final Examination :* *Maro A. Wells (2, 3, 4), Kathleen J. Harrison (2, 4), *Suzanne K. R. Clarke (2), *Elizabeth Preston-Thomas (2, 3, 4), Molly I. Govier (2), *A. J. Lee (2, 3), *A. H. Levy (2, 3), R. J. Carey (2), B. F. Vaughan (2), Margaret Ford (3, 4), Medora Storkey (1), P. C. Farrant (1), Patricia M. Gilbert (1), P. A. Normandale (1), J. P. Martin (1).

* Qualified. (1) Pathology. (2) Medicine. (3) Surgery. (4) Midwifery.

